# Building Trauma-Informed Approaches in Higher Education

**DOI:** 10.3390/bs12100368

**Published:** 2022-09-28

**Authors:** Lisa A. Henshaw

**Affiliations:** Wurzweiler School of Social Work, Yeshiva University, New York, NY 10033, USA; lisa.henshaw@yu.edu

**Keywords:** trauma-informed, culturally responsive, higher education institutions

## Abstract

Trauma-informed approaches serve as an essential framework for human service organizations and are now being applied in education settings, including higher education institutions (HEIs). The increasing incidence of traumatic events, including the global pandemic of COVID-19 and the systemic violence against persons of color, has prompted HEIs to examine how culture and inclusivity are conceptualized in the curriculum and reflected in institutional policy and programming. Within this context, there is a need to identify how trauma-informed approaches at HEIs can be culturally responsive. This article briefly summarizes evidence supporting the rationale for trauma-informed approaches at HEIs and how culture has historically been addressed through cultural competency and cultural humility. Cultural sensitivity and responsiveness are then conceptualized from a trauma-informed lens as informed by the literature. Finally, key concepts and theory relevant to applying culturally responsive trauma-informed approaches at HEIs are defined, with recommendations for policy, research, and practice.

## 1. Introduction

For the past 20 years, trauma-informed approaches have been applied to human service systems. Over time, the five core values of trauma-informed care conceptualized by Harris and Fallot [1]—safety, trustworthiness, choice, collaboration, and empowerment—have expanded to include peer support and cultural, historical, and gender issues [2]. A federally funded agency, the Substance Abuse and Mental Health Administration (SAMHSA) [2], has published guidelines for trauma-informed approaches that are nationally endorsed. While the sixth principle of trauma-informed approaches includes cultural, historical, and gender issues, further research is needed to understand how trauma-informed approaches can be culturally responsive in various institutional settings.

Federal initiatives and educational reform initially brought trauma-informed approaches into elementary and secondary schools [3,4,5]. More recently, higher education institutions (HEIs) with academic programs such as social work, nursing, education, and medicine [6,7,8,9] have adopted trauma-informed approaches to effectively meet students’ needs amidst the global pandemic, with cultural humility and responsiveness at the forefront following the cultural and collective trauma of George Floyd. For example, Goddard et al. [7] highlighted the student–educator relationship as being critical for supporting students’ stress regulation, suggesting that educators must embody empathy and support to create a safe base for students to learn. While research exists on the use of trauma-informed approaches in primary and secondary school systems, application at HEIs to date focuses on classroom teaching pedagogy.. Limited studies have explored the application of trauma-informed approaches in organizational systems at the broader level within HEIs.

This review will first, examine evidence that supports the application of trauma-informed approaches at HEIs. It will then summarize existing literature related to culturally responsive higher education. Lastly, key concepts and theory will be identified with recommendations to inform future research, policy, and practice. As indicated by Carello and Thompson [10], bringing trauma-informed approaches to HEIs requires acknowledging that in addition to students, educators and administrators also experience trauma, marginalization, and oppression. However, this article aims to focus on students. For the purpose of this article, the term persons of color is inclusive of Black, Latino, Indigenous persons, and Asian and Pacific Islanders.

### 1.1. Author Positionality

As recommended by Quiros [11], I approach the writing of this article from a trauma-informed lens and hence begin by locating myself. I am a white, Cisgender, able-bodied, middle class, straight, female, born and raised in the United States. My intersectionality is quite privileged, with experiences of oppression deriving solely from my gender identity. Being the child of an immigrant also informs my perspective of difference. As a junior faculty at a graduate school of social work and as a licensed clinical social worker, my educational frame of reference derives from social work. Due to my positionality, the knowledge that I have access to is situated culturally and academically amongst the majority group who hold more structural power; according to the American Council on Education and the U.S. Department of Education (ED), out of 700,000 full-time faculty at HEIs, 73.2% were white and 21.1% were faculty of color, with 3.1% being of international origin and 2.6% identified as having an unknown racial and ethnic background [12].

It is my aim, as an author, educator, social worker, critical ally, and human, to continuously engage in reflexive practice and critical thinking that transcend the boundaries of my privilege. This includes a commitment to questioning hegemonic structures and practices, including my own socialized assumptions and beliefs, and locating power structures and dynamics in the systems I take part in.

I consider myself a teacher/learner. My pedagogy is informed by the philosopher and educator Paulo Freire, coupled with my commitment to critical consciousness and action-oriented activism in the spaces I occupy. Freire’s philosophy includes intentionally attending to and neutralizing power dynamics in educational spheres, with a focus on dialogue and rejecting the banking method of education. This includes examining how personal and political factors interact and affect one’s work, with educators engaging students based on a relationship of mutual respect and neutrality [13].

### 1.2. Rationale for Culturally Responsive, Trauma-Informed Approaches in Higher Education

In higher education, the rationale for culturally responsive, trauma-informed approaches is multi-faceted. Research demonstrates that students may be exposed to trauma indirectly through the curriculum, field practicum, and while conducting research [14,15,16]. Whereas technology has made viewing traumatic events readily accessible for students contributing to individual exposures [17], advances in science have uncovered the neurobiological impact that trauma has on individual learning. Concentration, memory, executive functioning, information processing, language acquisition, and the socio-emotional aspects of learning may be affected, including class engagement with peers and instructors [18]. The human stress response to trauma, which includes numbing and/or dissociation, may be misinterpreted as lack of interest and disengagement in classroom activities and may impact behavioral challenges in field practicum related to attendance, tardiness, and overall disruption of working with clients [19].

The social environment is trauma-laden with COVID-19, racial violence and race-based trauma, active shooter events, and war [20,21]. Hate-based and race-based crimes against students’ cultural groups are increasing [22]. Disparities unearthed during the pandemic include COVID-19 illness and mortality rates, hate crimes based on experiences of discrimination and xenophobia, and learning [23].

COVID-19 prompted an upheaval at HEIs due to the unpredicted transition to remote learning. While the implications are still being explored, preliminary research shows significant disparities among students. For example, among UCLA students studying science, technology, engineering, and math (STEM), Barber et al. [24] found that among a sample of 947 respondents, underrepresented minority background (URM), first-generation, and low-income students were disproportionately affected due to expectations from them to help siblings at home with remote learning and to assist in addressing food insecurity and economic stress. Students’ learning was further impeded due to decreased social connectedness and sense of belonging amidst the global pandemic and lockdown—factors which have been shown to promote students’ psychosocial and academic outcomes [25].

Discrimination and xenophobia against Asian and Pacific Islander students were noted in the aftermath of COVID-19 due to the perceived location of the origin of the pandemic [26].COVID-19 mortality rates were shown to be higher among Black, African American, Latino, American Indian, Alaska Native, and Pacific Islander populations [27]. For example, during May 2020 in NYC, mortality rates for COVID-19 among Latinos were 187 per 100,000, and 184 per 100,000 among African Americans, in contrast to the mortality rate among White persons at 93 per 100,000 [27].

The data demonstrate an increase in traumatic event exposure in communities targeted by hate crimes since the onset of the pandemic. According to the U.S. Department of Justice and the U.S. Department of Health and Human Services, hate crime statistics gathered by the Federal Bureau of Investigation demonstrated an increase in hate crimes, in which 60% were directly related to race, ethnicity, or ancestry [28]. Alarmingly, there was a 30% increase in race-based hate crimes, with more than half (55%) of all race-based hate crimes driven by anti-Black bias [28]. Hate crimes against Asian individuals increased by more than 70% [28]. The second largest category of hate crimes were in reference to religion, comprising 15% of all hate crimes, and it was noted that conspiracy claims purporting that Jewish persons were spreading the coronavirus contributed to hate incidents [28].

## 2. Literature Review

### 2.1. The Role of Culture in Trauma-Informed Approaches

Culture may be defined as the values, beliefs, actions, customs, thoughts, communications, and behaviors of a racial, ethnic, religious, or social group [29,30]. Culture is included as the sixth principle in SAMHSA’s [2] guidelines for trauma-informed approaches under “cultural, historical and gender issues”. In their guidelines, SAMHSA asserts, The organization actively moves past cultural stereotypes and biases (e.g., based on race, ethnicity, sexual orientation, age, religion, gender identity, geography, etc.); offers access to gender responsive services; leverages the healing value of traditional cultural connections; incorporates policies, protocols, and processes that are responsive to the racial, ethnic and cultural needs of individuals served; and recognizes and addresses historical trauma [2] (p. 11).

Including this core principle represents an important paradigm shift in conceptualizing trauma-informed systems: cultural responsiveness throughout the human service organization is an explicit priority. Being “trauma-informed” means responding to the cultural needs of persons, including “issues” related to their intersectional identity, and considering historical traumas of the individual and/or cultural group [2].

The intersection of trauma and culture is well-documented. Traumatic event exposure occurs in the context of one’s culture, both the individual culture and the broader culture, and unique cultural differences influence how persons respond to trauma and perceive traumatic events [31]. According to Mohat et al. [32], historical trauma refers to a traumatic experience that is collective or experienced as a group, one that occurs over time and across generations. The nature of historical trauma is that the individual members of a group may experience trauma reactions in the present day although they were not present during the occurrence of the initial event. Thus, members of cultural groups are intrinsically connected through their shared history of trauma and resilience. Members of a cultural group can experience both external and internal reminders of historical trauma [33]. Public narratives through the media shape the dominant discourse, while lived experiences shape one’s personal experiences, which become intertwined. Thus, meaning that is ascribed to traumatic events and historical trauma becomes embedded in culture.

The power dynamics associated with historical traumas are entrenched. The core dynamics of trauma include disempowerment [34]; historical traumas that are large-scale atrocities and genocide, such as the Holocaust, slavery, etc., include narratives of oppression and disempowerment based on cultural identity. At the structural level among institutions, such as HEIs, these power dynamics may be reflected throughout system policies and practices. For example, the implicit and explicit curricula, and even the physical space of settings in terms of inclusivity. Trauma and culture may also intersect as part of one’s daily lived experience through individual interactions, including microaggressions that may trigger traumatic responses.

However, where power is stripped in the midst of trauma, whether individual or collective, there are also opportunities for resilience and growth, and trauma-informed approaches offer a unique opportunity for harnessing these strengths.

### 2.2. Moving Past Cultural Competence

The literature is heavily cloaked with many concepts that aim to define how to engage culturally diverse individuals and groups safely and equitably. Historically, HEIs have used the lens of cultural competence, and more recently, cultural humility. Though a systematic review of the literature is beyond the scope of this article, the following section reviews these concepts for clarification and historical context.

Cultural competence has been used for over 40 years to guide practitioners, educators, and policy makers in their process of engaging diverse populations. The concept has been explored, critiqued, and reimagined, replaced with concepts such as cross-cultural competence and multi-cultural competence. From a social work frame of reference, it has a storied history in the profession that is wrought with debate and criticism. As a concept that surfaced in the 1980s and developed over time as an essential ethical standard for social work practice, according to the National Association of Social Workers [30], cultural competence includes advocacy, activism, and “action to challenge institutional and structural oppression and the accompanying feelings of privilege and internalized oppression” (p. 10). In this framework, culture and diversity are often interchangeable in their meaning [35]. One point worthy of note is that NASW [30] operationalizes culture in alignment with SAMHSA’s sixth principle, in which culture encompasses the sociocultural lived experiences of individuals of different religion, gender, class, sexual orientation, age, and ability and is extended beyond race and ethnicity to include all aspects of intersectionality.

A major critique of cultural competence asserts that teaching cultural competence must go beyond understanding the experiences of oppressed groups and knowledge about persons who are “othered”; a critical examination of social structures and systematic oppressions, such as institutional racism, must also be included in order to avoid any implicit bias within the social work curriculum itself [36]. Additionally, the language of “competence” denotes expertise in another individual’s culture, which is a kind of mastery that is antithetical to the underlying meaning of cultural competence itself—social workers cannot master the “competence” of cultural groups through textbook learning, particularly the uniqueness of each individual within a cultural group [37]. 

A more up-to-date conceptualization of cultural competency in social work includes the process of social workers reflecting inward, examining their own culture—including cultural norms of socially constructed ideas related to race and other identities—and developing an awareness of personal and cultural bias [38]. It is also important to note social workers’ ethical obligation to cultural competence in the academic space, in which modeling for students is just as important as teaching in the curriculum.

### 2.3. Cultural Humility

The concept of cultural humility has recently been popularized and used in place of cultural competency, particularly in social work and other helping professions such as nursing and public health [39,40]. Cultural humility is reflexive in nature; it emphasizes self-refection and the development of awareness of one’s own power, privilege, and bias [41]. Gottlieb [42] purported that this entails a commitment to three major elements: (i) continuous self-reflection and self-awareness with the support of community, (ii) remaining open to learning and being taught about cultural difference from the standpoint of clients’ lived experiences versus how we have been instructed to define or come to understand them, and (iii) critically thinking and questioning the structural power dynamics (both power and privilege) that shape our lived realities and those of the people we work with (p. 465). Ultimately, applying the framework of cultural humility views the client as the expert and requires practitioners to consider how they may be inherently a part and a product of the very social systems we are challenging [42].

### 2.4. Cultural Sensitivity and Cultural Responsiveness in Trauma-Informed Approaches

Moving beyond cultural competence and cultural humility, this special issue explores the concepts of cultural responsiveness and cultural sensitivity within the framework of trauma-informed approaches. However, the concept of cultural sensitivity is used less frequently in the literature. It signifies an approach to professional practice that includes compassion for cultural differences and recognizing the role of culture in service delivery. It emphasizes attending to cultural nuances in a strengths-based manner in order to understand the cultural meanings of wellbeing [43].

In contrast, cultural responsiveness is widely discussed, frequently in the context of education and teaching pedagogy. Gay [44] defines cultural responsiveness as “using the cultural knowledge, prior experiences, frames of reference, and performance styles of ethnically diverse students to make learning encounters more relevant to and effective for them”. n applying cultural responsiveness to trauma-informed approaches, Blitz et al. [45] suggest being relationship-focused, harnessing the strengths of culture, and holding consideration for the intersection of structural oppressions and trauma. Cultural responsiveness offers space for individuals to feel culturally safe while involving them in the decision-making process [46] and asserts that knowledge is situated in historical, cultural, and social contexts [47].

Comeaux et al. [48] broadened their definition, adding the term transformative in their explication of cultural responsiveness, which this author finds herself to be most aligned with. Transformative cultural responsiveness (TCR) is defined as “an ongoing process of active learning and unlearning that leads to the capacity to recognize, understand, and effectively respond to the challenges and opportunities presented by the existence of cultural diversity of individuals and groups in different contexts and communities” [48] (p. 7). In the spirit of Freire, they suggested that TCR is a lifelong commitment to learning and unlearning, the development of critical awareness interlocking oppressions, attending to intersectionality, and moving beyond dialogue and reflection toward action [48].

## 3. Key Concepts and Theory to Guide Future Research and Practice

The following section highlights key concepts and theory that can be used to bridge culturally responsive trauma-informed approaches at HEIs. In order to build culturally responsive, trauma-informed programming and policy, there must be a common language and attention to discourse. How we communicate about trauma and the cultural nuances of trauma-informed approaches must be intentional.

### 3.1. Intersectionality

Culture is woven into an individual’s intersectional identity. Intersectionality refers to the interlocking nature of one’s social identities, including race, class, gender, sexual orientation, religion and ethnicity, age, ability, and nationality; it recognizes how these shape the privileges and oppressions that individuals may experience both individually and structurally [49]. Described by Crenshaw as a “prism for seeing the way in which various forms of inequality often operate together and exacerbate each other” [50], intersectionality is already embedded in the curriculum of many HEIs. Using an intersectional lens helps to name the power dynamics at play and to recognize the historical traumas embedded in cultural identity [51].

### 3.2. Critical Race Theory

Critical race theory has recently garnered attention in public spaces due to attempts by state legislatures and school boards to ban its teaching in the classroom in certain states in the U.S. [52]. This trend followed President Trump’s Executive Order to remove federal funding from diversity and inclusion training containing race and sex stereotyping or scapegoating [53]. Since the executive order, criticism has been building up due to the perception that CRT reprimands and places the blame on white persons for racism in the U.S., that CRT is unconstitutional and is disparaging, even discriminatory against white persons [54]. However, this author asserts that CRT as a theoretical framework for supporting social justice helps to achieve the opposite; rather than blaming one group, it seeks to analyze the power dynamics of institutions in order to identify and dismantle systematic racism.

While CRT centers race in this analysis, it also recognizes the intersection of race with all other elements of one’s intersectionality. At its core, CRT identifies race as a social construct and negates the notion that race is a biological construct. It recognizes the embeddedness and endemic nature of race in society through institutions, systems, and structures, as well as its interwovenness in public policy. CRT challenges the dominant ideology, including ideals of meritocracy, color blindness, and race neutrality, exposing how these claims are self-serving for groups in power to maintain the status quo [55].

### 3.3. Racial Trauma

Culturally responsive, trauma-informed approaches in HEIs require naming and responding to racial trauma in academic spaces. The Diagnostic and Statistical Manual for Mental Disorders (DSM-5) [56] does not specify race-based incidents in its diagnostic criteria [57]. Without a categorical structure for posttraumatic stress reactions based on raced-based experiences, the empirical evidence supporting the recognition and management of racial trauma remains scant. Due to this, racial trauma may even be underreported [58].

Comas-Diaz et al. [59] asserts that “racial trauma, a form of race-based stress, refers to People of Color and Indigenous individuals’ reactions to dangerous events and real or perceived experiences of racial discrimination” (p. 1). Racial trauma includes experiencing intimidation or threats, degradation or shaming, as well as observing these acts towards other persons of color, which can lead to dehumanization. Unique to racial trauma is the relentless re-exposure and collective nature of racism; while reactions to racial trauma mirror those to posttraumatic stress reactions, race-based trauma is both an individual’s experience and a shared experience by one’s cultural group.

### 3.4. Cultural Capital

Cultural capital is important to consider in culturally responsive, trauma-informed approaches at HEIs. It places cultural experiences at the center of analysis [60], and since it was developed by Bordieu, it has changed over time with various iterations. I follow Yosso’s [61] assertion that among students of color, culture can serve as a tool for nurturing and empowerment through communities of color. In this vein, cultural capital embodies the “knowledge, skills, abilities and networks” (p. 82) that students of color bring with them to school and that can be harnessed as resources, instead of being compared with the dominant culture (white, middle class communities) through a deficit-based lens. She purports that communities of color possess distinct cultural capital, which includes aspirational, familial, navigational, social, linguistic, and resistant capital. Expanding beyond communities of color, Pennell [62] suggests that a sixth form of cultural capital be included when conceptualizing queer cultural capital: transgressive capital. Transgressive capital highlights how historically marginalized communities, such as queer persons, are proactive in challenging boundaries that are oppressive, whether through institutional structures or socially constructed norms.

### 3.5. Critical Allyship

The framework of critical allyship suggested by Gates et al. [63] includes acknowledging biases, educating before engaging, extending the conversation beyond police brutality, targeting racism, and making critical allyship a research priority. The use of the term allyship has been corporatized and commodified. Gates et al. [63] reframed the concept as critical allyship, underscoring the connection to critical theory. Critical allyship embodies the commitment to “…interrogate experience and positionality within social relations of power and privilege” (p. 5). Similarly, Nixon [64] promotes principles of critical allyship as a vehicle for dismantling power differentials. To this end, critical allyship is associated with action and a lifelong pursuit and commitment, in which an element of sacrifice is recognized in an effort to dismantle oppressive systems.

### 3.6. Intentional Positive Disruption

According to Quiros [11], trauma-informed social work that embodies inclusion requires intentional positive disruption. While she applies this in the context of leadership, this framework and practice principle can be applied to create institutional cultures that foster critical thinking. This includes questioning what is considered as “normative” or “the status quo”. Use of intentional positive disruption has been echoed by Carello and Thomson [10] as an approach to trauma-informed work in academic spaces that centers equity and critically evaluates how actors and systems in education perpetuate oppression. Positively disrupting the dominant norms that perpetuate marginalization and oppression may take the form of dialogue, research, or teaching. While intentional positive disruption begins with locating one’s social position, it includes the examination of power relations within systems at the structural level.

## 4. Recommendations for Policy, Research, and Practice

The following recommendations are informed by the three areas in SAMHSA’s [2] guidelines for trauma-informed approaches: principles, assumptions, and domains. In addition to the six principles outlined by SAMSHA [2] discussed above, the four major assumptions offered as guidance for all trauma-informed policy and practice include: realizing the prevalence of trauma and how it affects persons; recognizing the signs and symptoms of trauma; responding by integrating knowledge about trauma and trauma recovery into policy and practice; and resisting retraumatization. Finally, the 10 domains SAMSHA [2] suggest institutions should focus on when implementing trauma-informed approaches comprise: governance and leadership; policy; physical environment; engagement and involvement; cross-sector collaboration; screening, assessment, and treatment services; training and workforce development; progress monitoring and quality assurance; financing; and evaluation.

### 4.1. Training and Education

Training and education must center on “moving beyond the performative” [11]. Despite the fact that diversity, equity, and inclusion trainings are trending, Ash et al. [65] assert that re-educating white leaders is necessary on an annual basis. Leaders must be willing to critically examine their own power and to be comfortable with sharing power [66]. The training and education process should focus on providing tools for self-interrogation and for locating oneself, as well as on how to recognize microaggressions, macroaggressions, and identity-based discrimination. It is critical that faculty and administrators learn the value of trauma-informed approaches as well as the effects of all forms of trauma on learning, including the lived experiences of racial trauma from a historical lens. Education and training should also include actionable steps and behaviors in order to be able to teach faculty and staff in the school community to shift from a deficit-based perspective to valuing cultural and community capital.

Tools must be offered to revise policy and programming as well as to teach members how to serve as a critical ally. As stated by Gates et al. [63], this entails going beyond the perception that serving as an ally can be “…an easily absorbed aspect of identity and being a good person” (p. 10), such as committing to “calling out” racism (p. 18). Similarly, Gray et al. [67] asserted that a “stop the line” approach should be implemented and incentivized. This approach supports individuals stepping forward to communicate any racist behavior, policy, or practice in academia, which this author recommends expanding to include the identification of any identity-based discrimination.

Program administrators and leaders at HEIs must buy into the value of trauma-informed approaches that are culturally responsive in order to model a top-down approach for faculty and staff. As asserted by SAMSHA [2], in the domains of leadership, training, and workforce development, investing in education and training on cultural responsivity within trauma-informed approaches is essential. This offers opportunities to implement trauma-informed principles of collaboration and mutuality, peer support, and empowerment among the school community that both faculty and students are able to benefit from.

### 4.2. Safety in the Physical and Virtual Space

Developing safety is an essential trauma-informed principle that supports transparency in communication and openness. SAMHSA’s [2] domain of the physical environment of the organization at HEIs includes not only the learning environment in the classroom and in zoom classes, but also through email communications and the physical space of the campus. For example, micro and macroaggressions, such as cultural racism, may be reflected in the manner an institution demonstrates preference for one cultural group over another [68]. Sue et al. [69] offered the example that “one’s racial identity can be minimized or made insignificant through the sheer exclusion of decorations or literature that represents various racial groups” (p. 274).

Openly communicating with transparency through program-wide emails about traumatic events in the environment that impact the school community promotes safety. It reflects that the HEIs realize the pervasive nature of trauma, recognizing its effects as being important enough to name and acknowledge. It also promotes trustworthiness and transparency through clearly discussing what resources are in place to offer support for emotional and physical safety, reinforcing that the community is physically safe. This embodies a trauma-informed approach, where individuals know what to expect. It fosters harnessing a sense of control and predictability, with the knowledge that persons impacted by trauma may be vulnerable to feeling triggered by interactions and communication where safety is not directly accounted for.

### 4.3. Policy and Curriculum

Promoting safety by reflecting sameness in the space is multifaceted. This can include individuals who occupy the space, such as the hiring of diverse faculty and administrators. Focusing on the domain of policy is necessary for the implementation of a trauma-informed approach that can be “hard wired” into practices and embedded within institutions such as HEIs [2] (p. 13). This intentional positive disruption is necessary to disrupt the status quo of “pervasive whiteness” [65] (p. 18).

HEIs should develop specific policies addressing equal opportunities among students for research and employment. Reducing otherness occurs when opportunities for speaking in class, research, adjunct teaching, and assistantships are inclusive of all identities. Curricula must be evaluated to ensure that courses reflect teachings that do not support a power differential of the status quo or a dominant norm. An evaluation of whether voices of historically marginalized communities are reflected in the curriculum must be conducted. This should include whether the curriculum has been created using a deficit or strengths-based lens.

### 4.4. Committees and Coalition Building

Carbado et al. [51] suggested that intersectionality be applied in unexplored places, highlighting how it can be used in coalition building and in developing alliances; we can recognize differences and identify commonalities. Including students of diverse intersectionality from historically marginalized communities in committees promotes empowerment, voice, and choice as well as collaboration and mutuality. This serves as intentional positive disruption to interrupt the norm of existing power differentials. Including students from historically marginalized communities in committees re-centers their voices from the margins.

Offering opportunities for students to come together through affinity groups or simply as a collective dialogue supports nearly all of SAMHSA’s [2] principles and assumptions for trauma-informed approaches. Offering space for affinity groups supports access to cultural capital and sharing lived experiences with students of diverse, intersectional identities, using intersectionality to promote connection and peer support. It empowers students with knowledge about their peers’ experiences, thus offering collaboration and mutuality. Coalitions can be built from these experiences, among faculty and students of diverse intersectionality, in order to neutralize the power differential and promote access to cultural capital.

### 4.5. Program Evaluation, Research, and Scholarship

Program evaluation is necessary to ensure ethical competence and to assess the outcome of the application of trauma-informed approaches that are implemented. One such measure is The Attitudes Related to Trauma-Informed Care (ARTIC) Scale. ARTIC is a previously established, psychometrically valid scale that has been modified for education settings [70]. It evaluates staff attitudes (favorable or unfavorable) toward the implementation of trauma-informed approaches. To date, the ARTIC scale has been used in education settings that include early education and K-12 school settings. Thus, program evaluation at the university level first requires conducting research that can validate the modification of ARTIC for HEI settings. Expanding the program evaluation to include qualitative measures, such as focus groups of students, further ensures progress monitoring and quality assurance. Exploring how students are impacted by the implementation of trauma-informed approaches is a necessary part of program evaluation. Including students in the research process offers opportunities for collaboration and mutuality and empowerment, voice, and choice.

This author further suggests that research be conducted to explore the lived experiences of historically marginalized students, serving as a needs assessment that evaluates identity-based discrimination on campuses. Conducting such an assessment aims to serve as an intentional positive disruption and critical allyship in order to expose the status quo and how it is experienced by all persons at HEIs. Qualitative research conducted by university faculty and students can employ a theoretical framework that is informed by CRT and intersectionality in order to ensure the centering of voices of historically marginalized communities.

CRT was originally developed to deconstruct race in law practices. However, it offers a valuable framework for exploring how the social construction of race impacts educational institutions. For example, Yosso et al. [55] asserted that CRT can be used to explore and analyze how “the social construct of race shapes university structures, practices, and discourses from the perspectives of those injured by and fighting against institutional racism” (p. 663). Following Quiros et al. [71], this author supports the application of CRT as a vehicle for challenging dominant narratives, particularly at HEIs, including the graduate schools for social work. Applying CRT ensures a commitment to ensuring that color blindness does not occur in the process of programming and policy making for students.

## 5. Discussion

With the increasing incidence of traumatic events, including COVID-19, racial violence, war, refugee crises, and active shooter events, trauma-informed approaches have proven beneficial in meeting students’ educational needs in the classroom as well as in field practicum, and in informing research [66]. While the professions of social work, public health, psychology, and nursing have long grappled with the concept of cultural competence, in curriculum and in practice [40,72,73], conceptualizing culture through the frame of trauma-informed approaches offers a new perspective, highlighting the intersection of trauma and culture by attending to power dynamics, history, and personal agency.

Across the literature, terms such as “critical” and “transformative” are used to expand the concept of cultural competency in order to be more inclusive and action oriented. From a social work frame of reference, this expansion is imperative; without action in policy and programming, there remains the risk of retraumatization. As the pandemic continues to unfold, students, faculty, and staff in higher education institutions endure the deleterious effects of chronic traumatic stress and loss. Teaching and learning in a shared trauma means that social work students are exposed to the same traumatic events as the clients that they are working with in their field placements [74]. Access to social media makes the viewing of traumatic events in the news and social media outlets readily available. Social media, while triggering, has contributed to accountability. That racial violence is happening every day and has an impact on students of color is an inescapable reality. Choosing to address that reality by supporting students at the institutions where they learn is an opportunity for support at the least, and for healing, at best.

This article aims to further the dialogue about trauma-informed practices at HEI.It specifically focuses on elements of cultural responsiveness through its historical review, identification of concepts and recommendations for application. Trauma-informed practices were intended for systems, and HEIs comprise large systems within institutions where power dynamics manifest at every level [75]. Menschner and Maul [76] highlighted how implementing trauma-informed approaches in healthcare systems “require a paradigm shift”(p. 10). The punctuated equilibrium of COVID-19 presents an opportunity for policy change at HEIs, for which research is needed to inform [77]. Thus, this serves as a call to action in higher education; a call to resist blissful ignorance and challenge history by naming trauma for what it is and addressing it, so that students can reach their full learning potential.

## Data Availability

Not applicable.
